# Hydrogen sulfide activatable metal-organic frameworks for Fluorescence Imaging-Guided Photodynamic Therapy of colorectal cancer

**DOI:** 10.3389/fbioe.2022.1032571

**Published:** 2022-10-07

**Authors:** Honghui Li, Mao Huang, Zixuan Wei, Jiawen He, Yunong Ma, Cuixia Lu, Albert Jin, Zhixiong Wang, Liewei Wen

**Affiliations:** ^1^ Guangdong Provincial Key Laboratory of Tumor Interventional Diagnosis and Treatment, Zhuhai People’s Hospital (Zhuhai Hospital Affiliated with Jinan University), Jinan University, Zhuhai, China; ^2^ Medical College, Guangxi University, Nanning, China; ^3^ Laboratory of Cellular Imaging and Macromolecular Biophysics, National Institute of Biomedical Imaging and Bioengineering, National Institutes of Health, Bethesda, MD, United States

**Keywords:** photodynamic therapy, metal-organic frameworks, hydrogen sulfide, colorectal cancer, fluorescence imaging

## Abstract

Photodynamic therapy (PDT) is a promising alternative and palliative therapeutic strategy for colorectal cancer (CRC). A novel photosensitizer with higher selectivity for CRC and fewer side effects is vital for clinical application. Given that the overexpression of hydrogen sulfide (H_2_S) in CRC, it is expected to provide a selective stimulus for activatable photosensitizers that in respond to the specific microenvironment. Herein, we report a novel development of metal-organic frameworks (MOFs) composed of meso-Tetra (4-carboxyphenyl) porphine (TCPP) and ferric ion (Fe^3+^) through a facile one-pot process. Experiments both *in vitro* and *in vivo* reveal that the MOF is capable of depredating in response to the high content of H_2_S in tumor microenvironment of CRC. Accompanying with the degradation and release of TCPP, the fluorescence and photosensitivity effect is switched from “off” to “on”, enabling the MOF to serve as a H_2_S activatable nano-photosensitizer for real-time fluorescence imaging-guided and targeted PDT of CRC.

## Introduction

Colorectal cancer (CRC) as a most common malignant tumors of digestive tract shows increasing trends in incidence globally and has been the third leading cause of cancer deaths worldwide (GBD 2019 Colorectal Cancer Collaborators, 2022). Although the treatment options and prognosis for CRC patients have improved through the development of surgical regimens and novel drugs, alternative therapeutic strategies are still urgently needed, especially for CRC patients with advanced cancers or who are intolerant to surgery or chemotherapy due to advanced age and other conditions such as cardiopulmonary insufficiency (Kawczyk-Krupka et al., 2016). Photodynamic therapy (PDT) with precise controllable, high spatially and temporally accuracy, and minimally invasiveness has been approved as adjuvant treatment approach for esophageal cancer, skin cancer and lung cancer (Li et al., 2020; Ma et al., 2022). The PDT is expected to become an alternative or palliative therapeutic strategy for CRC with the popularity of fiber enteroscopes/colonoscopies (Kaleta-Richter et al., 2019). Photosensitizers as critical components of PDT significantly affect the therapeutic efficacy. Nevertheless, clinical trials to date indicate that the drawbacks of traditional photosensitizers, such as low solubility in water, suboptimal selectivity in tumor and phototoxicity for normal tissues hinder the application of PDT for CRC (Yu et al., 2017; Wei et al., 2019; Xie et al., 2021). Therefore, it is highly desirable to develop novel photosensitizers with more tumor selectivity and less side effects, especially for certain cancer cell types.

Recently, various nano-photosensitizers with great water solubility, tumor selectivity have been developed. Firstly, the nano-photosensitizers are capable of accumulating in tumors through the enhanced permeability and retention (EPR) effect owing to their particle size and the abnormal vascular structure of tumors (Menon et al., 2013; Zhang et al., 2018; Li et al., 2021b). Secondly, the surface of nano-photosensitizers is modifiable with various targeting moieties to significantly improve the targeting efficacy (Zhong et al., 2015; Lin et al., 2021). Even more noteworthy are numerous smart nano-photosensitizers that activate the photosensitive effect in response to the certain tumor microenvironment (TME) (Wang et al., 2020; Liang et al., 2021; Pan et al., 2022). Interestingly, specific pathological features of TME including low pH, hypoxia, overexpressed enzymes, and redox conditions will enable the activatable nano-photosensitizers to release the photosensitizers upon the certain stimuli (Hao et al., 2020; Liu et al., 2020; Li et al., 2022; Ni et al., 2022). This approach can greatly increase the effective concentration of photosensitizers in tumor tissues, and reduce phototoxicity for normal tissues.

In addition to the microenvironment features that numerous tumors share, a unique physiological feature in CRC is the high expression of endogenous hydrogen sulfide (H_2_S) (Shi et al., 2018; Tao et al., 2019). A growing body of evidence suggests that the overexpression of cystathionine-β-synthase (CBS) promotes the H_2_S production in CRC to support tumor cell bioenergetics, proliferation, migration, and invasion (Szabo et al., 2013; Chen et al., 2022a). Accordingly, H_2_S can serve as a specific stimulator of the nanoplatform for selectively drug release, targeted imaging and therapy for CRC (Ma et al., 2017; Chen et al., 2019; Shi et al., 2019; Su et al., 2022). Hence, high content of H_2_S in CRC is also regarded as an endogenous stimulator for the smart nano-photosensitizers, which is expected to significantly improve the PDT efficacy of CRC and reduce the phototoxicity of normal tissues.

**SCHEME 1:**
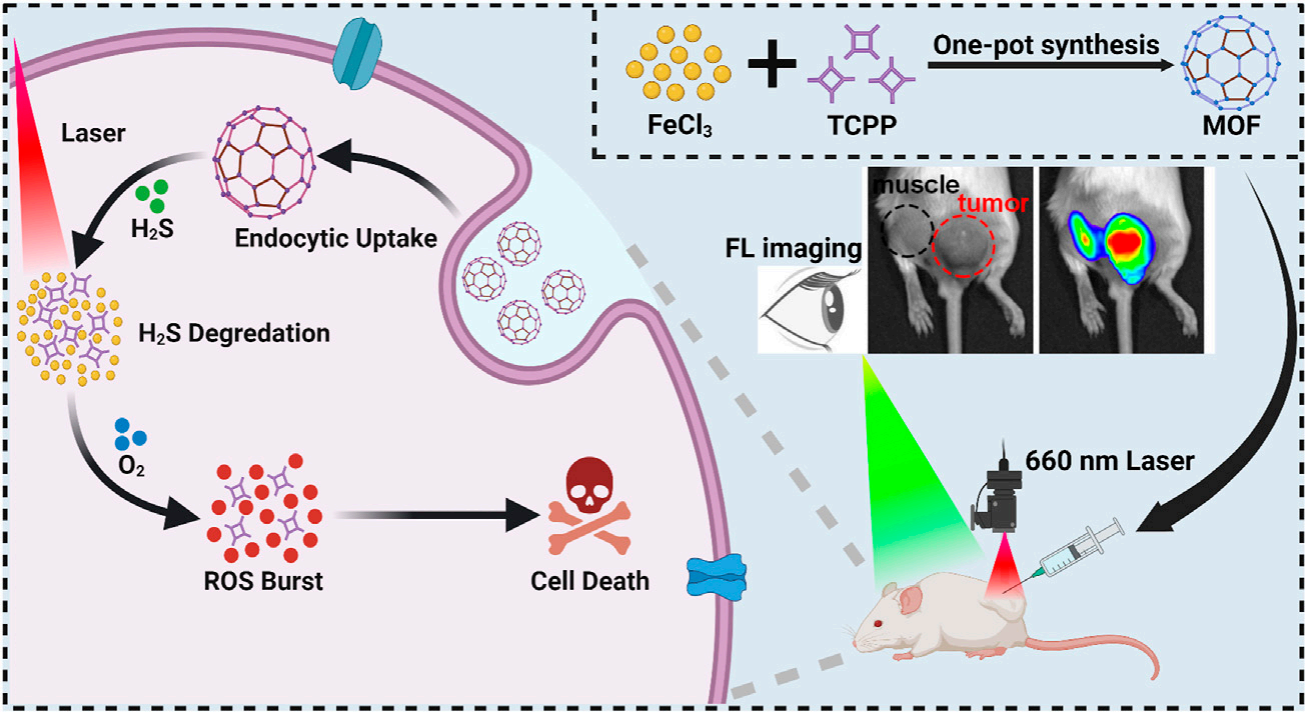
The illustration of MOF synthesis and schematic mechanism of MOF mediated fluorescence imaging-guided PDT.

In this study, a H_2_S-activatable metal-organic frameworks (MOFs) composed of a photosensitizer, meso-Tetra (4-carboxyphenyl) porphine (TCPP), and ferric ion (Fe^3+^) is prepared through a facile one-pot process for fluorescence imaging-guided PDT of CRC. After delivering to the TME of CRC, the coordination effect between Fe^3+^ and TCPP is disrupted owing to the conversion of Fe^3+^ to ferric iron (Fe^2+^) by excessive H_2_S, which results in the degradation of MOF and release of free TCPP. Meanwhile, the fluorescence and photosensitivity effect of TCPP is switched from “off” to “on” (Scheme 1). Consequently, this MOF provides a promising photosensitizer for real-time fluorescence imaging-guided PDT of colorectal cancer.

## Materials and methods

### Materials

FeCl_3_⋅6H_2_O and Meso-tetra (4-carboxyphenyl) porphine (TCPP) were purchased from Shanghai Aladdin Biochemical Technology Co., Ltd, Sodium hydrosulfide hydrate, N, N-Dimethylformamide (DMF) and dimethyl sulfoxide (DMSO) were acquired from Macklin Co., Ltd, 2′,7′-Dichlorodihydrofluorescein Diacetate (DCFH-DA) and Cell Counting Kit-8 were purchased from Med Chem Express Co., Ltd. Roswell Park Memorial Institute (RPMI) 1,640 medium, PBS (pH 7.4, 10 mm), trypsin (0.25% EDTA), fetal bovine serum (FBS), and antibiotics (penicillin-streptomycin, PS) were purchased from Gibco Life Technologies Co., Ltd.

### Characterization instruments

Morphological study and elemental mapping images of the MOF were performed using a transmission electron microscopy (FEI Talos F200X, FEI, Hillsboro, OR, United States). The hydrodynamic diameters of MOF were measured by dynamic light scattering (DLS, Malvern Zetasizer Nano-ZS ZEN 3600, Malvern Instruments, Worcester-shire, United Kingdom). UV-vis absorbance spectra and fluorescence spectra were determined with a microplate reader (Thermo Scientific, Waltham, MA, United States). The confocal images were observed by confocal laser scanning microscopy (CLSM, Leica TCS SP8, Leica Microsystems, Germany). H&E stains of mice tumors collected from different groups were imaged using an in-verted fluorescence microscope (DM IRE2, Leica, Wetzlar, Germany). Bioluminescence was detected using Living Image software (IVIS Lumina Series, PerkinElmer, Waltham, MA, United States).

### Metal-organic framework synthesis

Meso-tetra (4-carboxyphenyl) porphine (TCPP, 0.0988 g) and Iron (III) chloride hexahydrate (FeCl_3_.6H_2_O, 2.7301 g) were dissolved in 150 ml N, N-Dimethylformamide (DMF). After sonication for 5 min, the solution was added to a PTFE-lined Parr reaction vessel and reacted at 90°C for 6 h. At the end of the reaction, the collected solution was centrifuged (14,000 g, 10 min) to collect the precipitation, and the unreacted raw materials were washed away by DMF and ethanol. The centrifugation/resuspension process was repeated three times. The precipitate was re-suspended in ddH_2_O and the purified sample was lyophilized, yielding a purple-black powder.

### Degradation and ROS detection of metal-organic framework

The mixture of MOF and NaHS (ddH_2_O as a control group) was transferred to new EP tubes and placed on a flip agitator for 12 h. After the reaction, the reaction was centrifuged for 14,000 g, 10 min. After washing the supernatant, diluted MOF suspension in ethanol was freshly prepared for the DLS, TEM and fluorescence spectrum measurements. (Ex: 415 nm).

For ROS detection, the MOF suspension mixed with NaHS solution or ddH_2_O in equal proportion. Then, the solution was treated on a flip oscillator for 12 h. After centrifuged, the supernatant was removed. Subsequently, ABDA (100 μg ml^−1^) was added into a mixed solution. After irradiated with a 660 nm laser (50 mW cm^−2^), the absorption value at 378 nm was measured on nanodrop. The control group was a mixed solution without light. ABDA consumption (%) = (A_L_-A_0_)/A_D_*100%. A_L_ was the experimental group, A_0_ was the control group, and A_D_ indicated the absorption value of 100 μg ml^−1^ ABDA at 378 nm.

### ^1^O_2_ detection

After mixing MOF suspension with NaHS solution or ddH_2_O in equal proportion, the suspension was treated on a flip oscillator for 12 h, then centrifuged, the supernatant was removed, and then ABDA dissolved in DMSO was added into a mixed solution with a concentration of 50 μg ml^−1^ (TCPP: 25 μg ml^−1^ equivalent) and 100 μg ml^−1^ in ABDA, and irradiated under a 660 nm laser with an intensity of 50 mW cm^−2^. After the treatment is completed, the absorption value at 378 nm is measured on nanodrop. The control group was a mixed solution without light. ABDA consumption (%) = (A_L_-A_0_)/A_D_*100%. AL is the experimental group, A_0_ is the control group, and A_D_ is the absorption value of 100 μg ml^−1^ ABDA at 378 nm.

### Cellular uptake

CT26. WT cells and BNL. CL2 were inoculated into confocal plates for 24 h, then incubated with MOF concentration of 50 μg ml^−1^ for 6 h. After being washed with PBS for three times, the cells were observed under CLSM (Ex: 405 nm).

### Intracellular ROS detection

DCFH-DA was used to detect the intracellular ROS generation ability of MOF. Briefly, CT26. WT cells were inoculated into confocal plates for 24 h, then PBS and MOF were dissolved in RPMI 1640 medium and added in confocal dishes for 6 h. After washing with PBS for 3 times, the cells were incubated with 10 μm DCFH-DA fluorescent probe for 30 min, and then the light group was irradiated with 660 nm laser at 50 mW cm^−2^ for 5 min. Finally, the fluorescent dye was removed and the cells was washed by PBS twice. After washing with PBS, the intracellular fluorescence was monitored using CLSM (Ex: 488 nm).

### Cell viability assay

To evaluate cell compatibility of MOF, the BNL. CL2 cells were seeded into a 96-well plate for 24 h. Then, different concentrations of MOF were added into the plate and incubated for another 6 h. After washing with PBS, the cells were added CCK8 reagent for 1 h and the absorbance of 450 nm was detected by multi-function enzyme labeling instrument.

Additionally, the cytotoxicity *in vitro* was detected by CCK8 assay using CT26. WT cells. CT26. WT cells were seeded onto 96-well plates in 5% CO_2_ at 37°C for 24 h to allow the attachment of cells. Then, different concentrations of MOF were added into the plate and incubated for another 6 h. For the phototherapy groups, cells were exposed to the laser irradiation (660 nm, 50 mW cm^−2^, 5 min) after incubation with the samples. Then, 100 μL of CCK8 was added into each well, and the cells were incubated for another 2 h and the absorbance of 450 nm was read on a spectrophotometer.

### Tumor models

BALB/c mice (3–4 weeks old) were purchased from Zhuhai Bestone Biotechnology Co., Ltd, and all the reported animal experiments strictly complied with the regulations of the people’s Republic of China on the Administration of Experimental Animals and were approved by the Animal Ethics Committee of Guangxi University, P.R. China. To obtain CT26. WT tumor-bearing mice, mice were subcutaneously injected with 100 μL of 2 × 10^6^ CT26. WT cells to the right sides of each mouse.

### *In Vivo* fluorescence imaging

For H_2_S activated fluorescence of MOF in tumors, the 50 μL MOF (TCPP equiv 2.5 mg kg^−1^) solution was administrated by intratumoral and intramuscular injection, respectively. After that, fluorescence of the tumor was observed by using an IVIS imaging system. To study sample targeting and enrichment in the tumor site, 100 μL of MOF or TCPP solution (TCPP equiv 5 mg kg^−1^) was intravenously injected into the mice. Then the mice were imaged at several specific time points. After 24 h, mice were placed in a sealed plexiglas box for 5 min into which 3% isoflurane and 2 L min^−1^ oxygen flow was introduced for anesthesia, and sacrificed by cervical dislocation. Finally, the tumor and major organs collected from the sacrificed mice were imaged by an IVIS imaging system.

### Photodynamic therapy efficacy *in vivo*

On the first day when the tumor volume of CT26. WT tumor-bearing mice reached ∼100 mm^3^, the mice were randomly assigned into the following seven groups (six mice in each group): 1) Saline, 2) Saline + Laser, 3) TCPP + Laser 4) MOF and 5) MOF + Laser. The mice were intravenously administered with various preparations (equivalent TCPP concentration: 5 mg kg^−1^) *via* tail vein. At 4 h post-injection, only the light groups were irradiated with 660 nm laser (0.2 W cm^−2^, 5 min). The tumor volume and mice body weight were monitored every 3 days.

The tumor volume was measured according to the following equation: volume = length × width^2^ × 0.5. On the 15th day, the mice were placed in a plexiglas box for 5 min, during which 3% isoflurane and 2 L min−1 oxygen flow were introduced, then sacrificed by cervical dislocation and the tumors and organs were collected to using for H&E and Ki67 staining.

### Statistical analysis

Data were statistically analyzed by OriginLab (OriginLab Corporation, Northampton, MA) and GraphPad Prism eight software (GraphPad Software, Inc, La Jolla, CA, United States).

The *t*-test and one-way analysis of variance (ANOVA) were applied for significance analysis, followed by the Bonferroni test. *p* < 0.05 was considered to indicate a statistically significant difference.

## Results

### Characterization of metal-organic framework

**FIGURE 1:**
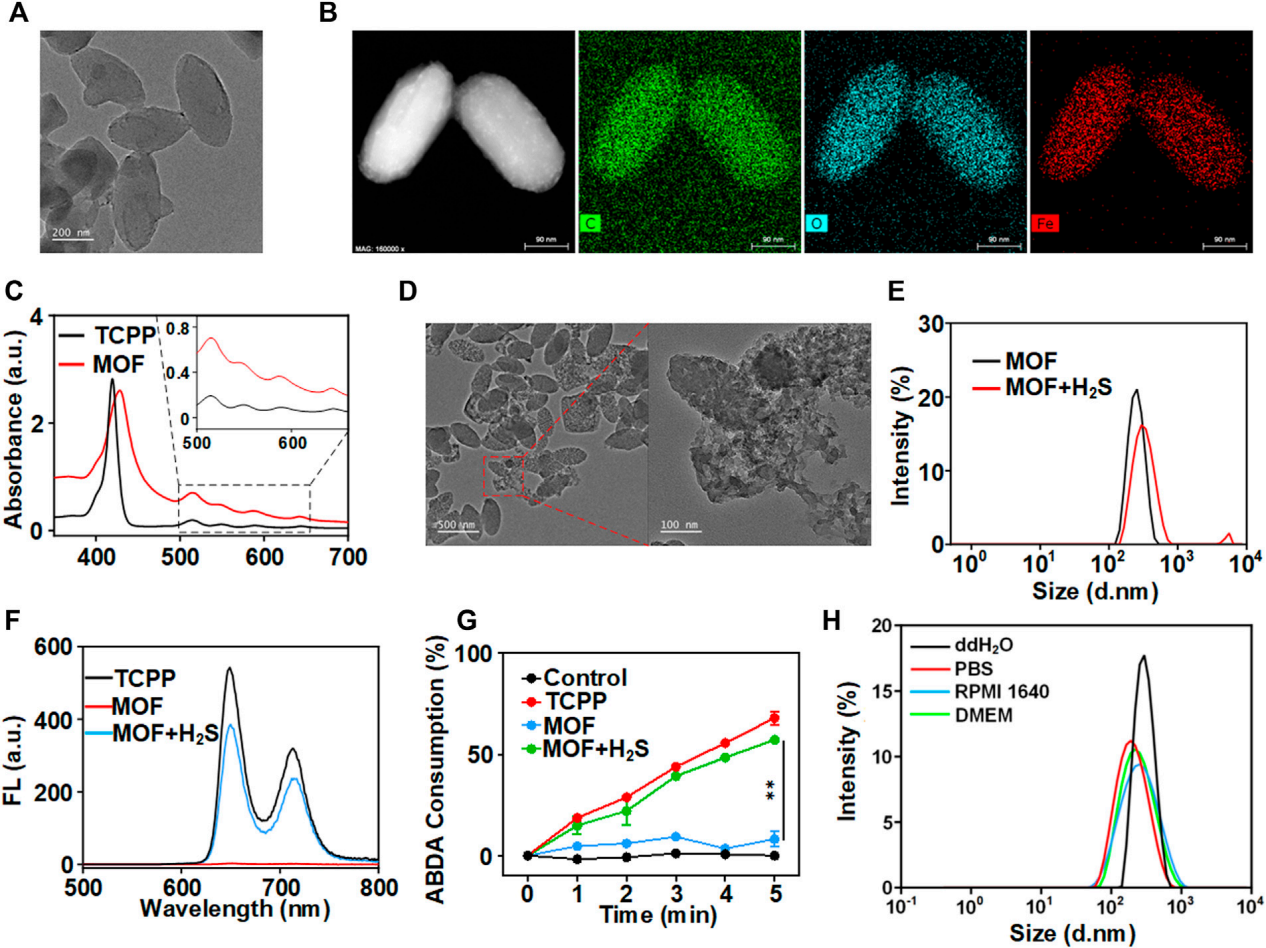
Characterization of MOF. **(A)** TEM image (scale bar: 200 nm). **(B)** Elemental mapping of MOF (scale bar: 50 nm). **(C)** UV−vis spectra of MOF and TCPP. **(D)** The TME of H_2_S-triggered bio-decomposition of MOF. **(E)** The hydrodynamic size of MOF with different conditions. **(F)** Fluorescence spectra of TCPP, MOF and MOF with NaHS. **(G)** Consumption rates of ABDA with different composites under 660 nm laser irradiation (50 mW cm^−2^) within 5 min **(H)** DLS measurement of MOF in different media.

Here, the facile synthesis of MOF could be realized through a one-pot solvothermal route by reacting TCPP and FeCl_3_.6H_2_O in a mixture of N, N-dimethylformamide (DMF) at 90°C for 6 h. A uniform nano-shuttle morphology of MOF with a dimension of approximately 250 nm in length and 100 nm in width was observed in transmission electron microscopy (TEM) images (Figure 1A). Moreover, a homogeneous distribution of the elements C, O and Fe could be detected through EDS-Mapping (Figure 1B). Then, the optical property of the MOF was investigated by UV-Vis absorption analysis. The four absorption peaks (516 nm, 550 nm, 591 nm and 645 nm) of MOF were consistent with the free ligand TCPP, suggesting the presence of photosensitizer TCPP moieties in the MOF (Figure 1C). The above results confirmed the successful preparation of MOF based on the coordination effect of Fe (III) and TCPP. The contents of TCPP in MOF was determined to be approximately 50% by weight from the standard UV-vis absorbance curve of TCPP and that of the MOF solutions after digested completely by concentrated hydrochloric acid. To clarify the role of H_2_S in activating MOF, sodium hydrosulfide (NaHS), as a classic H_2_S donor, was used to simulate endogenous H_2_S. After incubated with NaHS, the morphology of MOF changed and degraded into irregular fragments (Figure 1D). Meanwhile, the polydispersity index (PDI) of MOF increased from 0.038 to 0.209 with multiple peaks of size distribution (Figure 1E). Moreover, the fluorescence of MOF before and after incubation with H_2_S was detected. Obviously, there is only the negligible fluorescence of MOF owing to the aggregation induces quenching (ACQ). By contrast, under the H_2_S solution, the fluorescence of MOF recovered as the fluorescence of TCPP (Figure 1F). It confirmed the release of TCPP accompanying with the degradation of MOF. Then, the photosensitivity effect of MOF after activated by H_2_S was evaluated. A dye molecule, 9,10-Anthracenediyl-bis (methylene) dimalonic acid (ABDA) was used as a probe for detecting singlet oxygen (^1^O_2_). After pre-incubation with H_2_S, the ABDA absorption of MOF + H_2_S group significantly decreased along with the extension of laser irradiation time, and the trend is almost the same as that of the free TCPP (Figure 1G). In contrast, the ABDA absorption of MOF in absence of H_2_S showed negligible change comparing with the control (Figure 1G). Therefore, these results suggested that MOF would be degradation and release the TCPP in the microenvironment with high content of H_2_S. In addition to the fluorescence recovery, the photosensitivity of MOF would be also activated simultaneously, which implied the prospect of realizing fluorescence imaging-guided PDT.

The MOF stability was measured in the PBS, ddH_2_O and culture medium (Figure 1H). The hydrodynamic diameter of MOF in ddH_2_O, PBS, RPMI 1640 and DMEM were also approximately 300 nm. Moreover, the polydispersity index (PDI) showed that MOF in different media was between 0.136 and 0.238, indicating great monodispersity and general stability of MOF under physiologically relevant conditions before activation.

### Degradation and photosensitive effect of metal-organic framework *in vitro*

**FIGURE 2:**
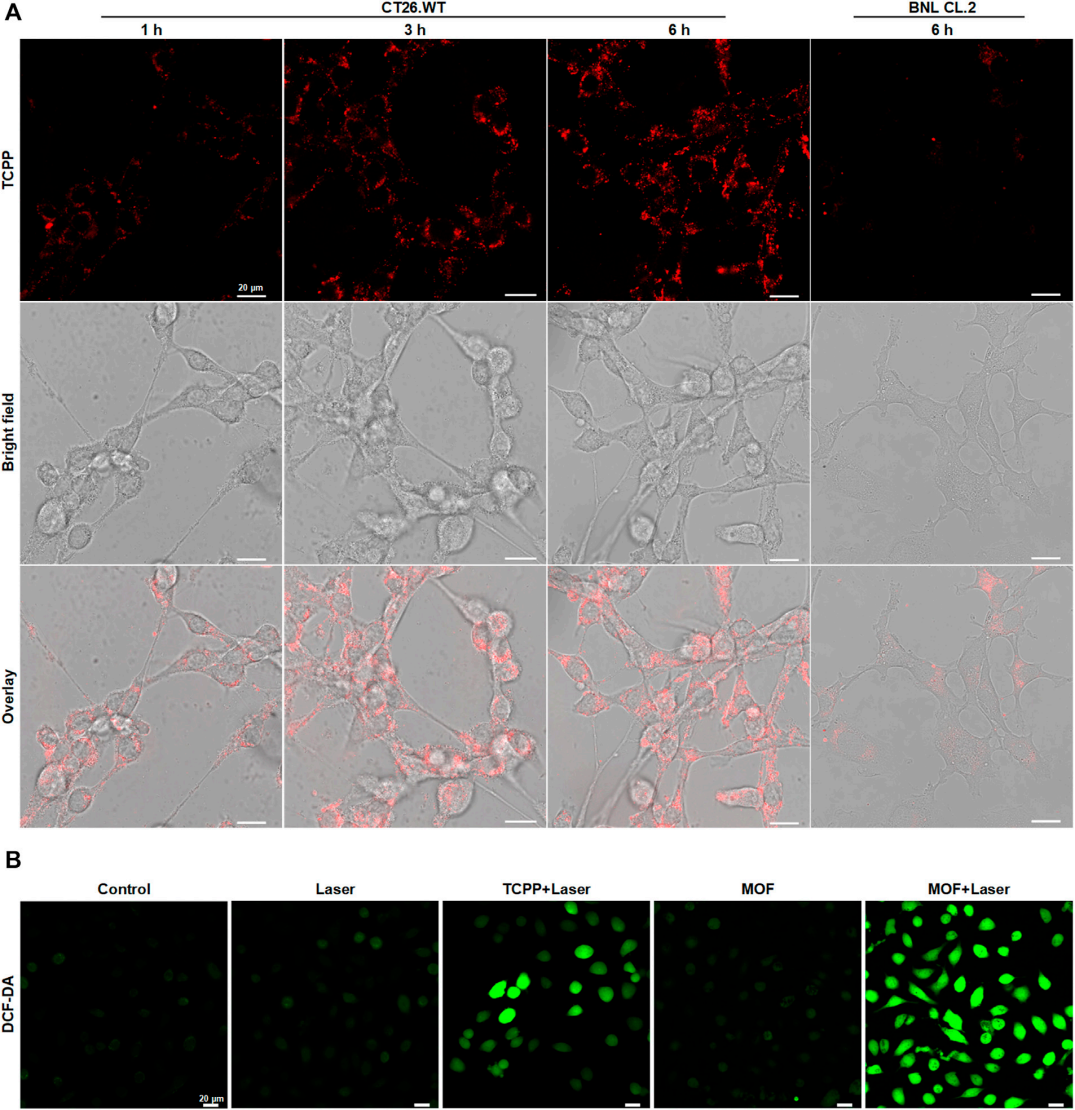
Degradation and photosensitive effect of MOF *in vitro* **(A)** Fluorescence changes of intracellular MOF by CLSM over time after different time of incubation. **(B)** Detection of cellular ROS generation by CLSM. The laser density was 50 mW cm^−2^ at 660 nm, and the irradiation time was 5 min. Scale bar: 20 μm.

High content of H_2_S (0.3–3.4 mmol L^−1^) as a unique feature of CRC played vital important role in the progression of CRC (Chen et al., 2022b). To investigate whether the MOF could be activated fluorescence and photosensitive effect in CRC cells, mouse colon carcinoma cells (CT26. WT) were used for study the cellular phagocytosis behavior of MOF firstly. The intracellular red fluorescence derived from TCPP gradually enhanced with the extension of incubation time, which demonstrated both the efficiently internalization of MOF by the CRC cells and the recovery of fluorescence triggered by high intracellular content of H_2_S (Figure 2A). By contrast, only faint fluorescence could be observed in the normal cells (BNL CL.2), indicating the fluorescence of MOF could be specifically switched from “off” to “on” in CRC cells. Accordingly, the activation of photosensitivity effect was further investigated by detecting the intracellular ROS. Benefiting from the better dispersion and more efficient cellular uptake of MOF than bare TCPP (Li et al., 2018), more bright fluorescence of H_2_DCF-DA was observed in MOF + Laser group than TCPP + Laser and others (Figure 2B). It heralded that the MOF was expected to be an efficient and specific photosensitizer for PDT of CRC.

### Metal-organic framework mediated photodynamic therapy *in vitro*

**FIGURE 3:**
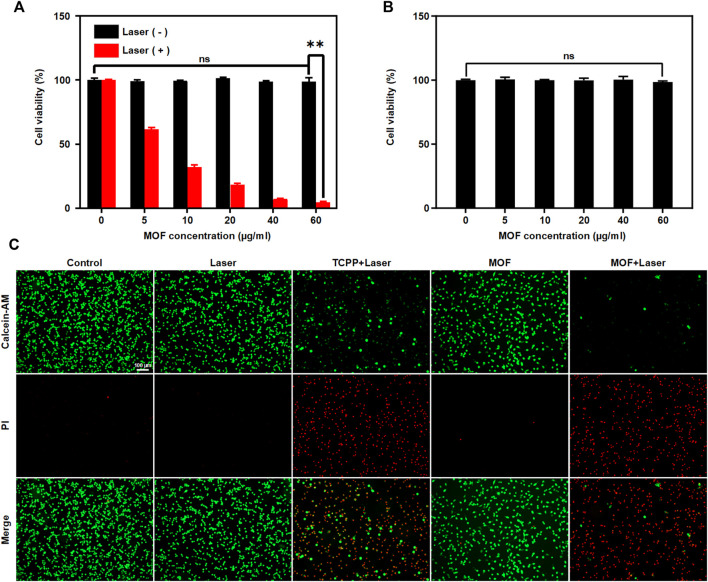
MOF mediated PDT *in vitro*. Cell viability of **(A)** CT26. WT cells and **(B)** BNL. CL2 cells treated with MOF with or without laser irradiation (660 nm, 50 mW cm^−2^, 5 min). **(C)** Detection of live/dead cells after various treatments. Live and dead cells were stained with calcein-AM (green) and PI (red), respectively. Scale bar: 100 μm. (**p < 0.01)

On the basis of the results above, MOF-induced cytotoxicity was assessed against CT26. WT cells. *In vitro* PDT efficiency was investigated by CCK8 assays. The MOF alone did not damage cells, exhibiting 100% cell viability even at 60 μg ml^−1^. Upon laser irradiation, the cell viability gradually decreased with increasing MOF contents. MOF can reach 60% of total cell killing at low concentrations (10 μg ml^−1^), revealing the highly-efficient PDT for cancer cells (Figure 3A). Then, the biosafety of MOF was explored *via* CCK8 assays, and BNL CL.2 cells were selected as the experimental cells. The MOF was harmless for normal cells, further indicating its good biocompatibility (Figure 3B). The therapeutic efficiency of the MOF was further confirmed by the Calcein-AM/PI double assay. The red fluorescence represented dead cells, while the green fluorescence represented live cells. Compared with other groups, the highest red/green ratio was observed for the MOF with laser irradiation treated group, implied the best therapeutic efficacy in MOF + Laser group (Figure 3C). It confirmed that neither normal cells nor the cancer cells viability affected by MOF in absence of light. And the excellent PDT efficacy of MOF ascribed to the activation of photosensitivity by the H_2_S-rich CRC microenvironment.

### Targeted fluorescence imaging of metal-organic framework

**FIGURE 4:**
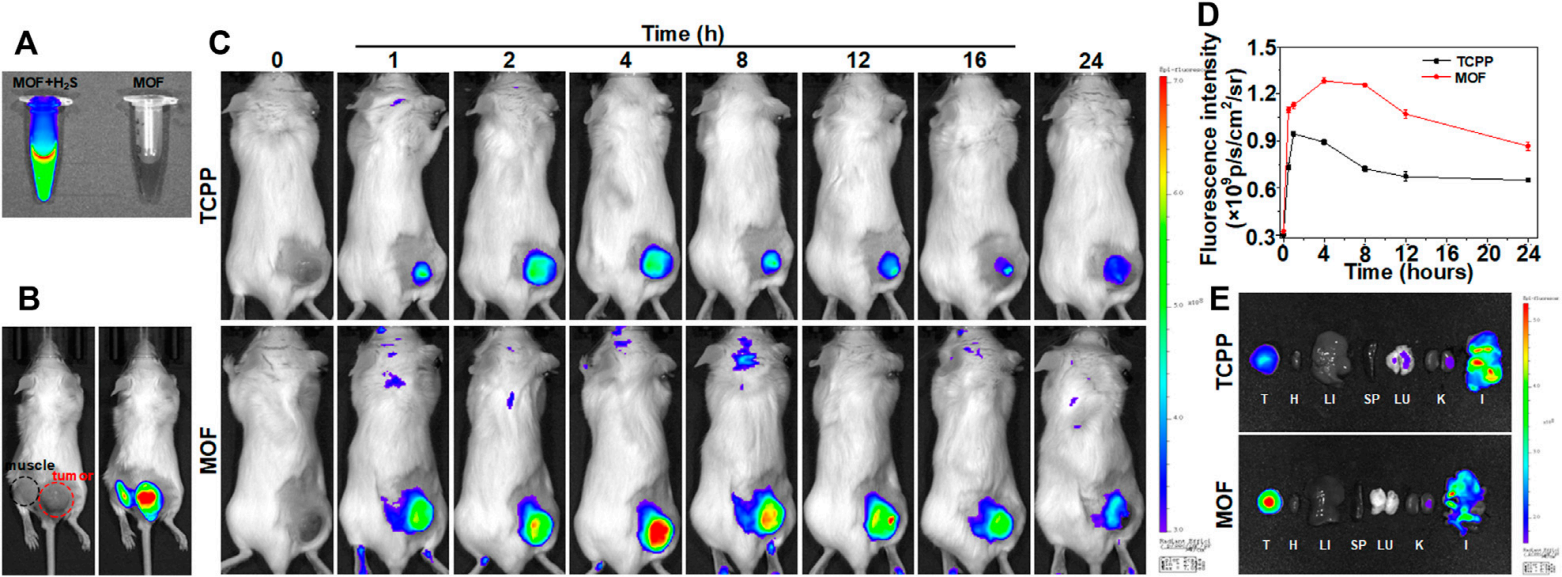
Targeted fluorescence imaging of MOF. **(A)** FL imaging of MOF and MOF with NaHS. **(B)** Fluorescence in tumor and muscle sites by intratumoral injection within 30 min. **(C)** Fluorescence imaging of mice over time by intravenous injection. **(D)** Relative fluorescence signal in tumor at different time points of tumor bearing mice after intravenous injection of MOF and TCPP in Figure 4C **(E)** *Ex vivo* fluorescence images of major organs and tumor at 4 h post-injection (λex = 675 nm, λem = 707 nm). (I: Intestine, H: Heart, LU: Lung, LI: Liver, K: Kidney, SP: Spleen, and T: Tumor).

The *in vivo* biodistribution of the MOF was investigated by fluorescence imaging. The mixture solution of H_2_S and MOF exhibited strong fluorescence with 675 nm exciting light, while no fluorescence was found in MOF alone (Figure 4A). Then, whether the fluorescence property of MOF could be specifically activated in the certain microenvironment of CRC was firstly investigated by observing the fluorescence at subcutaneous and intratumoral administration sites. After administration for 30 min, the tumor site showed bright fluorescence, while only the limited fluorescence signal could be observed in muscle (Figure 4B). It suggested that MOF-mediated fluorescence imaging could delineate the tumor regions and have the potential of serving as a targeted probe for monitoring and guiding the subsequent laser irradiation. Then, the distribution and accumulation of nano-photosensitizers were further verified after intravenous injected with MOF. The tumor site showed obvious fluorescence in both TCPP and MOF groups, and the fluorescence intensity for MOF group was higher than TCPP group (Figure 4C). Moreover, with the time extending, fluorescence signal was significantly increased and maintained for longer periods in the MOF group compared to TCPP group (Figure 4D). At 4 h post-injection, the *ex vivo* fluorescence images of major organs and tumors were statistical analysis. Brighter fluorescence of tumor was observed in MOF group than free TCPP group, suggesting a good targeting and selectivity of MOF for tumor (Figure 4E). In addition, the observed fluorescence signal in intestine (Figure 4E) implied that TCPP might metabolize through the excretion pathway faster than MOF before both being excreted *via* feces, consistent with the previous reports (Liu et al., 2017). These results suggested that MOF with great tumor selectivity, specificity, and retention capability was expected to provide a theranostic nano-photosensitizer for CRC. More importantly, MOF-mediated fluorescence imaging could provide temporally and spatially precise guidance for subsequent laser irradiation to remarkably enhanced PDT therapeutic effect and avoid normal tissue damage *in vivo*.

### Evaluation of photodynamic therapy efficacy and side effects

**FIGURE 5:**
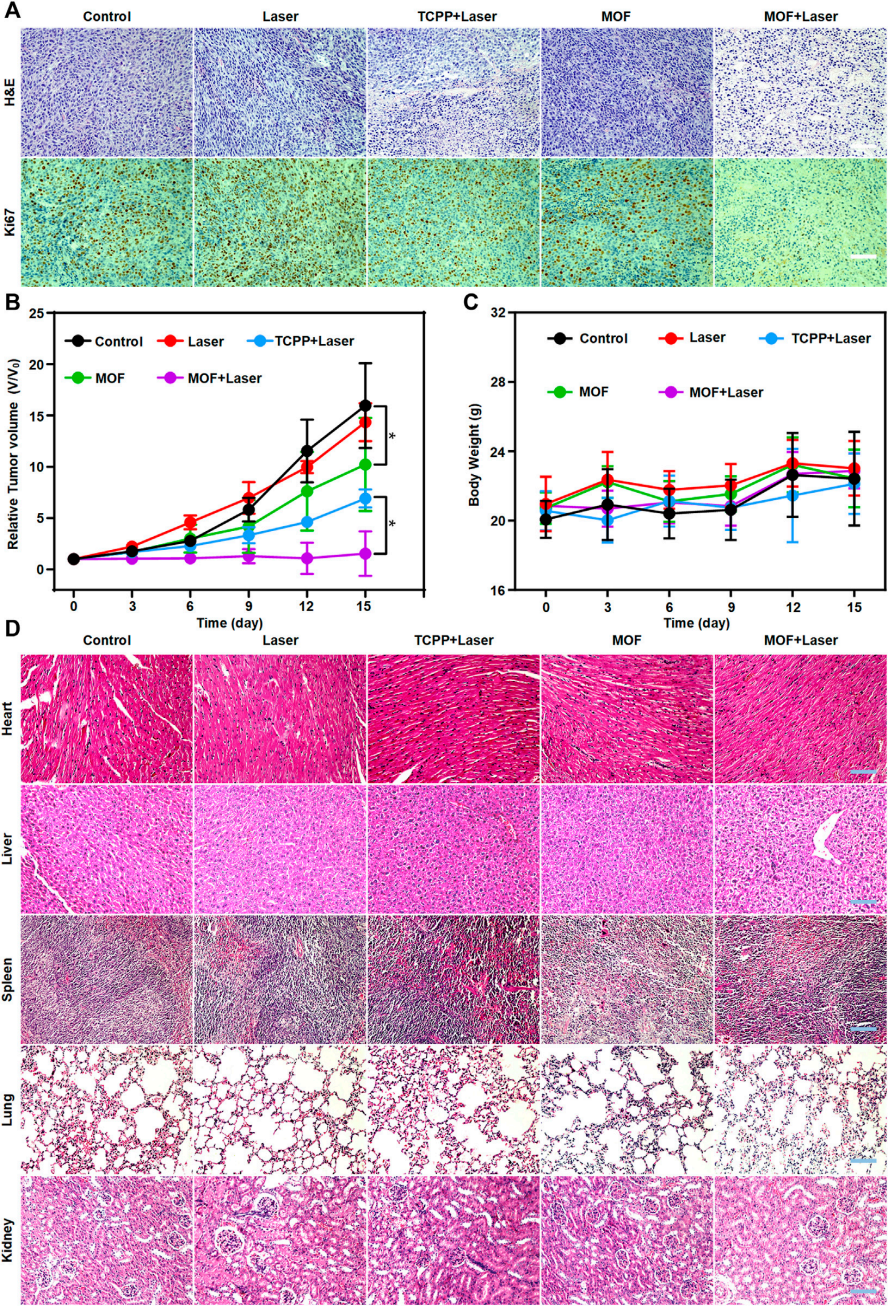
Evaluation of PDT efficacy and side effects. **(A)** H&E staining and Ki67 immunofluorescence of tumors. **(B)** Tumor volume Body weight and **(C)** Body weight of mice after various treatments during a 15-day treatment. **(D)** H&E staining of the five major organs (heart, liver, spleen, lung and kidney) with different treatments after 15 days. Scale bar: 100 μm. (*p < 0.05).

*In vivo* cancer inhibition of PDT based on MOF was evaluated by intravenous treatment of mice bearing subcutaneous CT26. WT tumors. The mice were divided into five groups. After different treatment, the tumor slices were verified by H&E and Ki67 immunofluorescence staining. Prominent cell necrosis and abundant karyorrhectic debris was observed in MOF + Laser group (Figure 5A). The tumor volumes were recorded after different treatment. It is worth noting that the tumor growth also was moderately inhibited in MOFs only group, presumably owing to the weak oxidative damage induced by the daily light in animal facility. According to the change of tumor volumes during treatment process, the tumor growth of MOF + Laser group was remarkably inhibited, and the efficacy of MOF-mediated PDT was also better than the TCPP + Laser group (Figure 5B). Next, the biosafety of MOF-mediated PDT was analyzed. The body weights of all treatment mice showed no distinct reductions with the increasing treatment time, indicating the negligible toxicity and side effects (Figure 5C). Furthermore, the biosafety of MOF was confirmed by H&E staining of main organs excised from different groups. The histopathology slices of major organs showed negligible morphological change, implying negligible systemic toxicity (Figure 5D). Therefore, the photosensitivity effect of MOF could be specifically activated in the certain CRC microenvironment, and MOF as a H_2_S activatable nano-photosensitizer was expected to highly-efficiently suppress tumor growth while avoiding side effects for normal tissues under laser irradiation.

## Discussion

CRC is the third most common cancer worldwide, and more than 50% of patients will develop to colorectal cancer liver metastases due to the absence of early symptoms and untimely diagnosis (Hernandez-Alejandro et al., 2022). Surgical resection alone is regarded as the standard of care for patients with liver metastases, but the recurrence rates remain high (Nordlinger et al., 2008). PDT is originally developed as an adjuvant therapy that enhanced the effect of surgery nearly 29 years ago (Dahlman et al., 1983). It has been approved by the Food and Drug Administration to treat pre-cancerous skin lesions of the face or scalp as early as in 1999 (Ch, 2012). Although PDT has not yet been approved to serve as a standard treatment for CRC, recent experimental data demonstrated that patients with early colon cancer presents a remarkable prolongation of survival under the treatment with PDT (Hodgkinson et al., 2017). Therefore, PDT as an adjuvant therapy is promising for CRC.

The enteroscopes are essential tools for CRC localization and examination (Sun et al., 2016). Nevertheless, the diagnoses based on enteroscopy often exist bias due to the lack of the clear boundaries between cancerous and normal tissues in the white-field and judgment of the tumor foci through naked eye. Thus, for precision treatment of CRC, more accurate imaging techniques are needed to assist with PDT. In the recent studies, imaging modalities such as fluorescence imaging and photoacoustic imaging are used to guide solid tumor treatments with high sensitivity and excellent contrast (Li et al., 2021a; Wang et al., 2021). In particularly, the specific contrast agents mediated novel imaging techniques can provide a more reliable alternatives for preoperative diagnosis and intraoperative navigation of CRC.

Recurrence and metastasis are remained the main challenge for CRC therapy, especially for the patients with unresectable CRC tumors. Fortunately, earlier studies indicated that PDT-treated cells induced immunogenic cell death (ICD) by releasing and danger-associated molecular patterns (DAMPs), and elicits antitumor immune response by activating CD8^+^ T cell expansion and function (Jeong et al., 2021). In the previous studies, nano-photosensitizers loaded CPG (immunologic adjuvant) can efficiently boost the host antitumor immuno-responses and achieve long-term therapeutic effects (Cai et al., 2020). In addition, immune checkpoint blockade therapy has gained recognition as promising approaches to overcome various cancers in the past decade. The combination of immune checkpoint inhibitors and PDT presented superior synergistic efficacy in diverse cancers such as triple-negative breast cancer and non-small cell lung cancer (Duan et al., 2016). It is worth noting that monoclonal antibodies (mAb) against programmed death receptor-1 (PD-1), pembrolizumab and nivolumab have shown considerable activity in patients with advanced colorectal cancer with DNA mismatch repair deficient/microsatellite instability-high (dMMR/MSI-H) tumors (Chen et al., 2020). Therefore, it is a potentially therapeutic direction to creating a nanoplatform to integration of photosensitizers and immunological agents, which will afford systemic antitumor effects by combination with immunotherapy and PDT.

## Conclusion

In this study, a H_2_S activatable nano-photosensitizer (MOF) was developed to precise and highly efficient PDT of CRC. The fluorescence and photosensitivity effect of MOF was quenched in storage and during blood circulation due to ACQ. Only if the MOF accumulated in the tumor regions of CRC, the fluorescence and photosensitivity effect was “turn on”, accompanying with the degradation and release of free photosensitizer (TCPP). Firstly, it was capable to serve as a specific fluorescence imaging probe of CRC to delineate tumor regions, which was important for precise planning the irradiation area and reducing the phototoxicity of normal tissues. Secondly, as a H_2_S activatable nano-photosensitizer, it was very suitable for PDT of tumors including CRC and breast cancer, etc., and it was promising for PDT of certain tumor. Last but not least, as a novel photosensitizer with convenient synthesis, simple composition and good biological safety, it had the potential of clinical translation.

## Data Availability

The original contributions presented in the study are included in the article/Supplementary Material, further inquiries can be directed to the corresponding authors.

## References

[B1] Cai Z. Xin F. Wei Z. Wu M. Lin X. Du X. (2020). Photodynamic therapy combined with antihypoxic signaling and CpG adjuvant as an *in situ* tumor vaccine based on Metal–Organic framework nanoparticles to boost cancer immunotherapy. Adv. Healthc. Mat. 9 (1), 1900996. 10.1002/adhm.201900996 31746153

[B41] Ch S. K. (2012). Photo dynamic therapy in oral diseases. Int. J. Biol. Med. Res. 3 (2), 1875–1883.

[B43] Chen E. X. Jonker D. J. Loree J. M. Kennecke H. F. Berry S. R. Couture F. (2020). Effect of combined immune checkpoint inhibition vs best supportive care alone in patients with advanced colorectal cancer: The Canadian Cancer Trials Group CO. 26 Study. JAMA oncol. 6 (6), 831–838. 32379280 10.1001/jamaoncol.2020.0910 PMC7206536

[B2] Chen J. Xue F. Du W. Yu H. Yang Z. Du Q. (2022a). An endogenous H2S-activated nanoplatform for triple synergistic therapy of colorectal cancer. Nano Lett. 22 (15), 6156–6165. 10.1021/acs.nanolett.2c01346 35852844

[B3] Chen J. Xue F. Du W. Yu H. Yang Z. Du Q. (2022b). An endogenous H2S-activated nanoplatform for triple synergistic therapy of colorectal cancer. Nano Lett. 22, 6156–6165. 10.1021/acs.nanolett.2c01346 35852844

[B4] Chen W. Ni D. Rosenkrans Z. T. Cao T. Cai W. (2019). Smart H2S-Triggered/Therapeutic system (SHTS)-Based nanomedicine. Adv. Sci. (Weinh). 6 (22), 1901724. 10.1002/advs.201901724 31763153 PMC6864508

[B40] Dahlman A. Wile A. G. Burns R. G. Mason G. R. Johnson F. M. Berns M. W. (1983). Laser photoradiation therapy of cancer. Cancer Res. 43 (1), 430–434. 6847782

[B42] Duan X. Chan C. Guo N. Han W. Weichselbaum R. R. Lin W. (2016). Photodynamic therapy mediated by nontoxic coreshell nanoparticles synergizes with immune checkpoint blockade to elicit antitumor immunity and antimetastatic effect on breast cancer. J. Am. Chem. Soc. 138 (51), 16686–16695. 27976881 10.1021/jacs.6b09538 PMC5667903

[B5] GBD 2019 Colorectal Cancer Collaborators (2022). Global, regional, and national burden of colorectal cancer and its risk factors, 1990-2019: a systematic analysis for the global burden of disease study 2019. Lancet Gastroenterol. Hepatol. 7 (7), 627–647. 10.1016/S2468-1253(22)00044-9 35397795 PMC9192760

[B6] Hao Q. Wang Z. Zhao W. Wen L. Wang W. Lu S. (2020). Dual-responsive polyprodrug nanoparticles with cascade-enhanced magnetic resonance signals for deep-penetration drug release in tumor therapy. ACS Appl. Mat. Interfaces 12 (44), 49489–49501. 10.1021/acsami.0c16110 33079514

[B7] Hernandez-Alejandro R. Ruffolo L. I. Sasaki K. Tomiyama K. Orloff M. S. Pineda-Solis K. (2022). Recipient and donor outcomes after living-donor liver transplant for unresectable colorectal liver metastases. JAMA Surg. 157, 524. 10.1001/jamasurg.2022.0300 35353121 PMC8968681

[B8] Hodgkinson N. Kruger C. A. Abrahamse H. (2017). Targeted photodynamic therapy as potential treatment modality for the eradication of colon cancer and colon cancer stem cells. Tumour. Biol. 39 (10), 101042831773469. 10.1177/1010428317734691 28990490

[B9] Jeong S. D. Jung B. K. Ahn H. M. Lee D. Ha J. Noh I. (2021). Immunogenic cell death inducing fluorinated mitochondria‐disrupting helical polypeptide synergizes with PD‐L1 immune checkpoint blockade. Adv. Sci. (Weinh). 8 (7), 2001308. 10.1002/advs.202001308 33854870 PMC8025002

[B10] Kaleta-Richter M. Kawczyk-Krupka A. Aebisher D. Bartusik-Aebisher D. Czuba Z. Cieslar G. (2019). The capability and potential of new forms of personalized colon cancer treatment: Immunotherapy and Photodynamic Therapy. Photodiagnosis Photodyn. Ther. 25, 253–258. 10.1016/j.pdpdt.2019.01.004 30611864

[B11] Kawczyk-Krupka A. Bugaj A. M. Latos W. Zaremba K. Wawrzyniec K. Kucharzewski M. (2016). Photodynamic therapy in colorectal cancer treatment--The state of the art in preclinical research. Photodiagnosis Photodyn. Ther. 13, 158–174. 10.1016/j.pdpdt.2015.07.175 26238625

[B12] Li H. Zhang Y. Liang L. Song J. Wei Z. Yang S. (2022). Doxorubicin-loaded metal-organic framework nanoparticles as acid-activatable hydroxyl radical nanogenerators for enhanced chemo/chemodynamic synergistic therapy. Mater. (Basel) 15 (3), 1096. 10.3390/ma15031096 PMC8838206 35161041

[B13] Li J. Wang A. Zhao L. Dong Q. Wang M. Xu H. (2018). Self-assembly of monomeric hydrophobic photosensitizers with short peptides forming photodynamic nanoparticles with real-time tracking property and without the need of release *in vivo* . ACS Appl. Mat. Interfaces 10 (34), 28420–28427. 10.1021/acsami.8b09933 30067331

[B14] Li X. Lovell J. F. Yoon J. Chen X. (2020). Clinical development and potential of photothermal and photodynamic therapies for cancer. Nat. Rev. Clin. Oncol. 17 (11), 657–674. 10.1038/s41571-020-0410-2 32699309

[B15] Li Y. Ye F. Zhang S. Ni W. Wen L. Qin H. (2021a). Carbon-Coated magnetic nanoparticle dedicated to MRI/photoacoustic imaging of tumor in living mice. Front. Bioeng. Biotechnol. 9, 800744. 10.3389/fbioe.2021.800744 34926438 PMC8675129

[B16] Li Y. Zhou R. Xiao D. Shi S. Peng S. Wu S. (2021b). Polypeptide uploaded efficient nanophotosensitizers to overcome photodynamic resistance for enhanced anticancer therapy. Chem. Eng. J. 403, 126344. 10.1016/j.cej.2020.126344

[B17] Liang L. Wen L. Weng Y. Song J. Li H. Zhang Y. (2021). Homologous-targeted and tumor microenvironment-activated hydroxyl radical nanogenerator for enhanced chemoimmunotherapy of non-small cell lung cancer. Chem. Eng. J. 425, 131451. 10.1016/j.cej.2021.131451

[B18] Lin L. Song X. Dong X. Li B. (2021). Nano-photosensitizers for enhanced photodynamic therapy. Photodiagnosis Photodyn. Ther. 36, 102597. 10.1016/j.pdpdt.2021.102597 34699982

[B19] Liu H. Yao J. Guo H. Cai X. Jiang Y. Lin M. (2020). Tumor microenvironment-responsive nanomaterials as targeted delivery carriers for photodynamic anticancer therapy. Front. Chem. 8, 758. 10.3389/fchem.2020.00758 33134254 PMC7550754

[B20] Liu W. Wang Y. M. Li Y. H. Cai S. J. Yin X. B. He X. W. (2017). Fluorescent imaging‐guided chemotherapy‐and‐photodynamic dual therapy with nanoscale porphyrin metal–organic framework. small 13 (17), 1603459. 10.1002/smll.201603459 28244202

[B21] Ma Y. Li X. Li A. Yang P. Zhang C. Tang B. (2017). H2 S-activable MOF nanoparticle photosensitizer for effective photodynamic therapy against cancer with controllable singlet-oxygen release. Angew. Chem. Int. Ed. 56 (44), 13752–13756. 10.1002/anie.201708005 28856780

[B22] Ma Y. Xiao F. Lu C. Wen L. (2022). Multifunctional nanosystems powered photodynamic immunotherapy. Front. Pharmacol. 13, 905078. 10.3389/fphar.2022.905078 35645842 PMC9130658

[B23] Menon J. U. Jadeja P. Tambe P. Vu K. Yuan B. Nguyen K. T. (2013). Nanomaterials for photo-based diagnostic and therapeutic applications. Theranostics 3 (3), 152–166. 10.7150/thno.5327 23471164 PMC3590585

[B24] Ni W. Li Y. Liang L. Yang S. Zhan M. Lu C. (2022). Tumor microenvironment-responsive nanodrug for clear-cell renal cell carcinoma therapy via triggering waterfall-like cascade ferroptosis. J. Biomed. Nanotechnol. 18 (2), 327–342. 10.1166/jbn.2022.3250 35484753

[B25] Nordlinger B. Sorbye H. Glimelius B. Poston G. J. Schlag P. M. Rougier P. (2008). Perioperative chemotherapy with FOLFOX4 and surgery versus surgery alone for resectable liver metastases from colorectal cancer (EORTC intergroup trial 40983): a randomised controlled trial. Lancet 371 (9617), 1007–1016. 10.1016/s0140-6736(08)60455-9 18358928 PMC2277487

[B26] Pan W. L. Tan Y. Meng W. Huang N. H. Zhao Y. B. Yu Z. Q. (2022). Microenvironment-driven sequential ferroptosis, photodynamic therapy, and chemotherapy for targeted breast cancer therapy by a cancer-cell-membrane-coated nanoscale metal-organic framework. Biomaterials 283, 121449. 10.1016/j.biomaterials.2022.121449 35247637

[B27] Shi B. Ren N. Gu L. Xu G. Wang R. Zhu T. (2019). Theranostic nanoplatform with hydrogen sulfide activatable NIR responsiveness for imaging-guided on-demand drug release. Angew. Chem. Int. Ed. 58 (47), 16826–16830. 10.1002/anie.201909883 31532051

[B28] Shi B. Yan Q. Tang J. Xin K. Zhang J. Zhu Y. (2018). Hydrogen sulfide-activatable second near-infrared fluorescent nanoassemblies for targeted photothermal cancer therapy. Nano Lett. 18 (10), 6411–6416. 10.1021/acs.nanolett.8b02767 30239208

[B29] Su Z. Kong L. Dai Y. Tang J. Mei J. Qian Z. (2022). Bioresponsive nano-antibacterials for H_2_S-sensitized hyperthermia and immunomodulation against refractory implant–related infections. Sci. Adv. 8 (14), eabn1701. 10.1126/sciadv.abn1701 35394829 PMC8993125

[B30] Sun B. Li W. Liu N. (2016). Curative effect of the recent photofrin photodynamic adjuvant treatment on young patients with advanced colorectal cancer. Oncol. Lett. 11 (3), 2071–2074. 10.3892/ol.2016.4179 26998124 PMC4774444

[B31] Szabo C. Coletta C. Chao C. Modis K. Szczesny B. Papapetropoulos A. (2013). Tumor-derived hydrogen sulfide, produced by cystathionine-beta-synthase, stimulates bioenergetics, cell proliferation, and angiogenesis in colon cancer. Proc. Natl. Acad. Sci. U. S. A. 110 (30), 12474–12479. 10.1073/pnas.1306241110 23836652 PMC3725060

[B32] Tao C. An L. Lin J. Tian Q. Yang S. (2019). Surface plasmon resonance-enhanced photoacoustic imaging and photothermal therapy of endogenous H2 S-triggered Au@Cu2 O. Small 15 (44), e1903473. 10.1002/smll.201903473 31513347

[B33] Wang B. Dai Y. Kong Y. Du W. Ni H. Zhao H. (2020). Tumor microenvironment-responsive Fe(III)-Porphyrin nanotheranostics for tumor imaging and targeted chemodynamic-photodynamic therapy. ACS Appl. Mat. Interfaces 12, 53634–53645. 10.1021/acsami.0c14046 33205657

[B34] Wang Z. Zhan M. Li W. Chu C. Xing D. Lu S. (2021). Photoacoustic cavitation‐ignited reactive oxygen species to amplify peroxynitrite burst by photosensitization‐free polymeric nanocapsules. Angew. Chem. Int. Ed. Engl. 60 (9), 4770–4781. 10.1002/ange.202013301 33210779

[B35] Wei W. Jiang D. Ehlerding E. B. Barnhart T. E. Yang Y. Engle J. W. (2019). CD146‐Targeted multimodal image‐guided photoimmunotherapy of melanoma. Adv. Sci. (Weinh). 6 (9), 1801237. 10.1002/advs.201801237 31065511 PMC6498137

[B36] Xie J. Wang Y. Choi W. Jangili P. Ge Y. Xu Y. (2021). Overcoming barriers in photodynamic therapy harnessing nano-formulation strategies. Chem. Soc. Rev. 50 (16), 9152–9201. 10.1039/d0cs01370f 34223847

[B37] Yu X. Gao D. Gao L. Lai J. Zhang C. Zhao Y. (2017). Inhibiting metastasis and preventing tumor relapse by triggering host immunity with tumor-targeted photodynamic therapy using photosensitizer-loaded functional nanographenes. ACS Nano 11 (10), 10147–10158. 10.1021/acsnano.7b04736 28901740

[B38] Zhang D. Wen L. Huang R. Wang H. Hu X. Xing D. (2018). Mitochondrial specific photodynamic therapy by rare-earth nanoparticles mediated near-infrared graphene quantum dots. Biomaterials 153, 14–26. 10.1016/j.biomaterials.2017.10.034 29096398

[B39] Zhong J. Wen L. Yang S. Xiang L. Chen Q. Xing D. (2015). Imaging-guided high-efficient photoacoustic tumor therapy with targeting gold nanorods. Nanomedicine Nanotechnol. Biol. Med. 11 (6), 1499–1509. 10.1016/j.nano.2015.04.002 25933697

